# Bacterial and fungal isolation from face masks under the COVID-19 pandemic

**DOI:** 10.1038/s41598-022-15409-x

**Published:** 2022-07-18

**Authors:** Ah-Mee Park, Sundar Khadka, Fumitaka Sato, Seiichi Omura, Mitsugu Fujita, Kazuki Hashiwaki, Ikuo Tsunoda

**Affiliations:** Department of Microbiology, Kindai University Faculty of Medicine, 377-2 Ohnohigashi, Osakasayama, Osaka 589-8511 Japan

**Keywords:** Microbiology, Public health

## Abstract

The COVID-19 pandemic has led people to wear face masks daily in public. Although the effectiveness of face masks against viral transmission has been extensively studied, there have been few reports on potential hygiene issues due to bacteria and fungi attached to the face masks. We aimed to (1) quantify and identify the bacteria and fungi attaching to the masks, and (2) investigate whether the mask-attached microbes could be associated with the types and usage of the masks and individual lifestyles. We surveyed 109 volunteers on their mask usage and lifestyles, and cultured bacteria and fungi from either the face-side or outer-side of their masks. The bacterial colony numbers were greater on the face-side than the outer-side; the fungal colony numbers were fewer on the face-side than the outer-side. A longer mask usage significantly increased the fungal colony numbers but not the bacterial colony numbers. Although most identified microbes were non-pathogenic in humans; *Staphylococcus epidermidis*, *Staphylococcus aureus*, and *Cladosporium*, we found several pathogenic microbes; *Bacillus cereus, Staphylococcus saprophyticus*, *Aspergillus*, and *Microsporum*. We also found no associations of mask-attached microbes with the transportation methods or gargling. We propose that immunocompromised people should avoid repeated use of masks to prevent microbial infection.

## Introduction

The rapid global spread of severe acute respiratory syndrome coronavirus 2 (SARS-CoV-2) and the resulting coronavirus disease 2019 (COVID-19) pandemic have led to urgent efforts to prevent the viral transmission. The most traditional and reasonable method to prevent respiratory infections is to wear face masks; several research groups have demonstrated its effectiveness against the respiratory viral transmission before the COVID-19 pandemic^1,2^. During the COVID-19 pandemic, increasing lines of evidence have supported the effectiveness of wearing face masks against SARS-CoV-2 and the droplets^3,4^. However, the World Health Organization (WHO) claims that face masks are effective only when used with hand hygiene, the proper use, and disposal of masks^5^.

**Figure 1:**
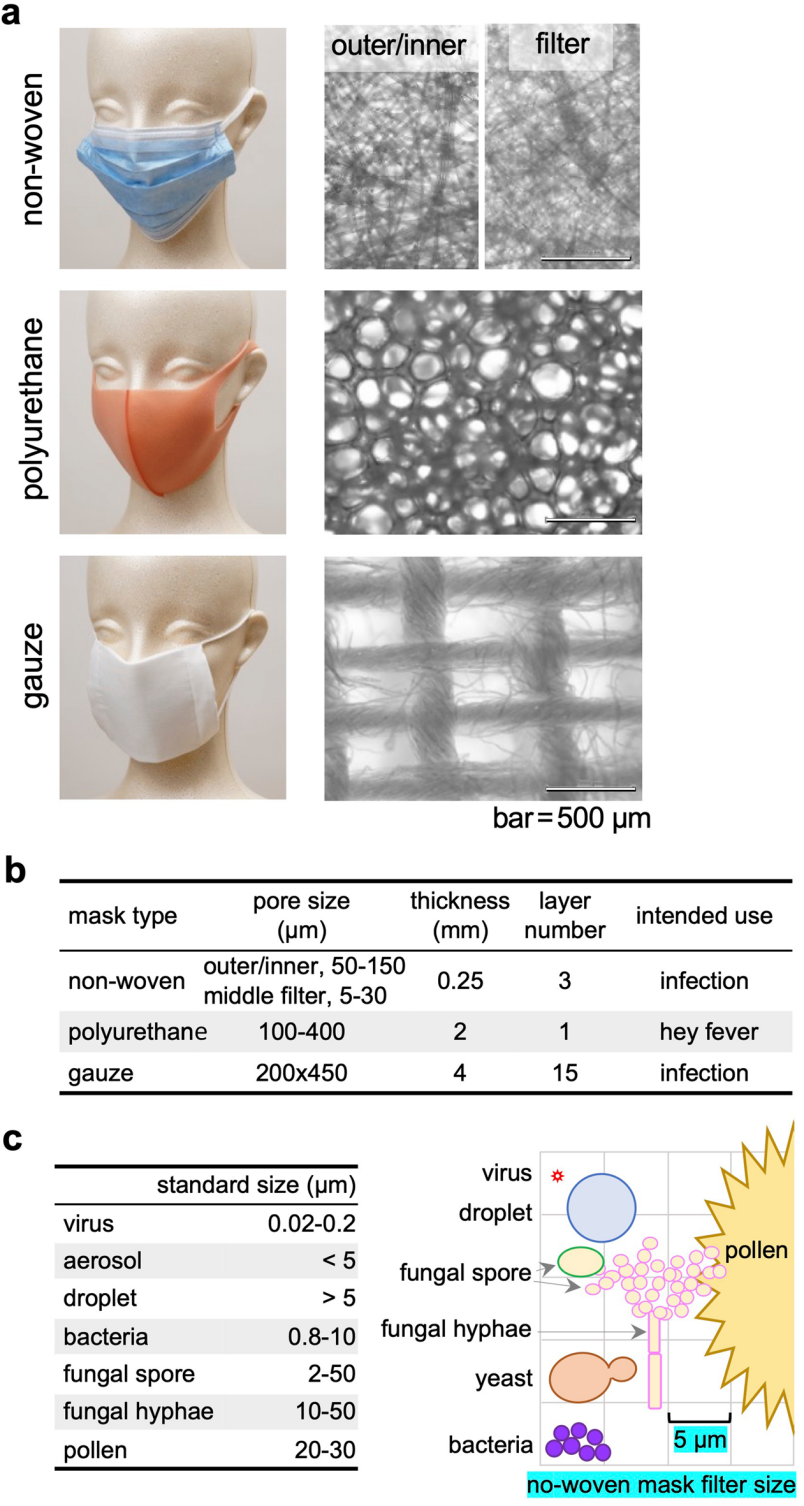
Face mask types and the sizes of microbes. (**a**) Macroscopic and microscopic images of three different types of face masks that are commercially available. Non-woven masks have three layers: the pore size of the outer and inner layers are identical (50–150 µm); the pore size of the middle layer (considered as a filter) is smaller (5–30 µm). Microscopic images were taken by the Olympus Microscope CX33 with the CCD Camera DP22 (bar = 500 µm). (**b**) Pore size, thickness, layer, and intended use of three mask types. The pore size of face masks from manufacturers’ instruction was confirmed using the microscopic images shown in (**a**) (right panels). (**c**) The standard size of microbes and particles (left panel) and their comparisons with the pore size (5 µm) of the middle filter of non-woven masks (right schema).

Three types of face masks are commercially available for daily lives in Japan: (1) non-woven, (2) polyurethane, and (3) gauze or cloth masks (Fig. 1a,b). Non-woven masks are commonly used worldwide to prevent droplet infections by most respiratory microbes, including SARS-CoV-2 (Fig. 1c). Polyurethane masks have been used to protect against hay fever, particularly in Asian countries. Since polyurethane masks are easy to breathe and washable, the masks have become popular and have been reused several times during the COVID-19 pandemic. Although gauze masks are less popular, the masks can be washed, reused, and effectively prevent infections. Thus, the Japanese government distributed gauze masks to all citizens because of the shortage of non-woven masks during the early stage of the COVID-19 pandemic.

Although the effectiveness of face masks against viral transmission has been extensively studied^3,4^, the hygiene issues in mask usage remain unclear. The standard mask usage is disposable non-woven masks. In some cases, however, people may use non-woven masks repeatedly or use different types of masks in different situations depending on their socioeconomic cultures. For example, in Japan, the short supply of non-woven masks led to the repeated use of disposable non-woven masks and the use of other types of face masks, such as handmade masks and polyurethane masks^6^. Even after the shortage of mask supply has been resolved, some people have used disposable non-woven masks repeatedly or other types of face masks.

Among environmental pathogens, viruses cannot replicate without infecting host cells; most bacteria and fungi can survive and grow on various materials depending on the conditions. Bacteria and fungi are widely present on the surface of the materials used in our daily lives (e.g., currency notes and in public transportation systems), where we can detect pathogenic bacteria and fungi^7–10^. Although a few studies reported bacterial or viral contamination on masks in experimental and clinical settings^11–13^, there has been no study on what and how many both bacteria and fungi adhere to masks used daily in community setting bases; this is the neglected hygiene issue under the COVID-19 pandemic. Since masks can be a direct source of infection to the respiratory tract, digestive tract, and skin, it is crucial to maintain their hygiene to prevent bacterial and fungal infections that can exacerbate COVID-19. Thus, in this study, following a survey of 109 volunteers on their mask usage and lifestyles, we aimed to quantify and identify the bacteria and fungi attached to the face masks by culturing microbes isolated from the masks.

## Results

### Mask types, gender differences, and duration of mask usage

**Figure 2:**
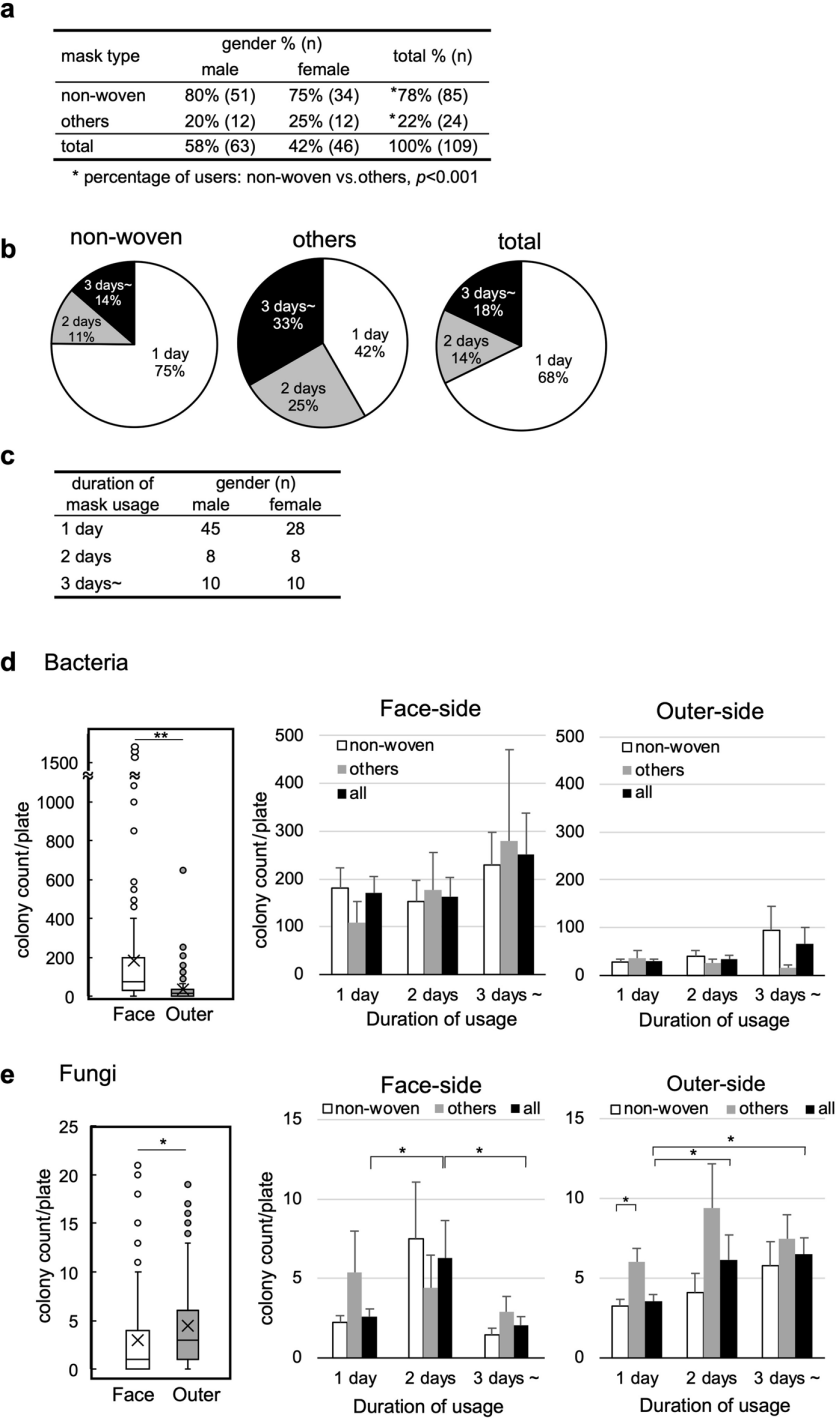
Survey results of the mask usage and microbe colony counts on the face-side and outer-side of the face masks. (**a**) Usage of non-woven masks and other mask types (others) among male and female participants (n = 109). Most “others” were polyurethane masks except a few gauze or cloth masks. (**b**) Duration of usage in non-woven, other mask types, and total (non-woven and others combined). The percentage of “others” wearing the same masks for two days or more (58%) was significantly higher than that of non-woven mask users (25%, *P* < 0.001). (**c**) Duration of mask usage in each gender (no significant difference). (**d**,**e**) Bacteria (**d**) and fungi (**e**) on the face-side and outer-side masks were cultured separately after pressing each mask surface onto agar plates. Microbial colony counts/plate (left panels); in boxplots, the cross symbols, bars, and dots indicate the mean, median, and outliers, respectively. Microbial colony counts on the face-side (middle panels) and outer-side (right panels) were compared based on the mask types and duration of mask usage. Mean + standard error of the mean (SEM). The paired *t*-test and Student’s *t*-test were used for statistical analyses. *P < 0.05; **P < 0.001.

Although the numbers of COVID-19 patients were relatively low in Japan during the study period, most people wore face masks in public places, and all survey participants wore face masks. First, we collected information about the mask types and duration of mask usage from 109 participants: 63 male (58%) and 46 female (42%). The majority (78% in total) of the participants used non-woven masks (Fig. 2a); the percentage of the non-woven mask users was significantly higher than that of the other mask type users (*P* < 0.001, most of them were polyurethane mask users except a few gauze or cloth mask users). Regarding the duration of mask usage, we found that 75% of non-woven mask users wore the masks for a single day. In contrast, 58% of the other mask type users wore the same masks for two days or more (Fig. 2b). This could be because other mask types, including polyurethane, gauze, and cloth masks, are designed washable for repeated usage; the users commonly washed and reused their masks multiple times. On the other hand, we found no significant differences between genders regarding the mask types and usage duration (Fig. 2a,c).

### Microbial counts on the face-side and outer-side of masks

Microbes on the masks were cultured by pressing the face-side and outer-side of the masks onto agar plates (two plates per participant: the face-side and outer-side). We incubated the agar plates for 18 hours (h) and 5 days for bacterial and fungal propagation, respectively, and conducted colony counting.

Bacteria (Fig. 2d): We observed bacterial colonies in 99% of the samples on the face-side and 94% on the outer-side; no colony was seen in one sample on the face-side and six samples on the outer-side. The colony counts of the face-side and outer-side were 168.6 ± 24.7 and 36.0 ± 7.0 [mean ± standard error of the mean (SEM)], respectively. We compared the colony counts between the face-side and outer-side in each individual and found that the mean colony counts were 13.4-times higher on the face-side of masks (paired *t*-test, *P* < 0.001). To evaluate the influence of the mask types and duration of mask usage, we compared the colony counts among those who used the mask for one day (3–6 h), two days, and longer based on the mask types [non-woven, others, and all (non-woven and others combined)]. We found no significant differences in the colony counts among the different mask types, regardless of the duration of usage.

Fungi (Fig. 2e): We observed fungal colonies in 79% of the samples on the face-side and 95% on the outer-side. The colony counts of fungi were fewer than those of bacteria and the colony counts on the face-side and outer-side were 4.6 ± 1.9 and 6.1 ± 1.9 (mean ± SEM), respectively. In contrast to the bacterial colonies, the fungal colony counts in each individual were 2.4-times higher on the outer-side than on the face-side (paired *t*-test, *P* < 0.05). When the participants used the same masks for more than two days, the fungal colony counts were increased on the outer-side of masks, compared with the one-day usage. There were no statistical differences in the colony counts between non-woven and “others” mask users except for the fungal colony counts of the outer-side of masks after one-day usage.

Since females preferentially make up their faces, we examined whether the bacterial and fungal colony counts could be different between males and females. Only the bacterial colony counts in the face-side samples of one-day users were significantly different, lower in females (Fig. S1).

### Microbial colonies and lifestyles: gargling, transportation, and natto consumption

**Figure 3:**
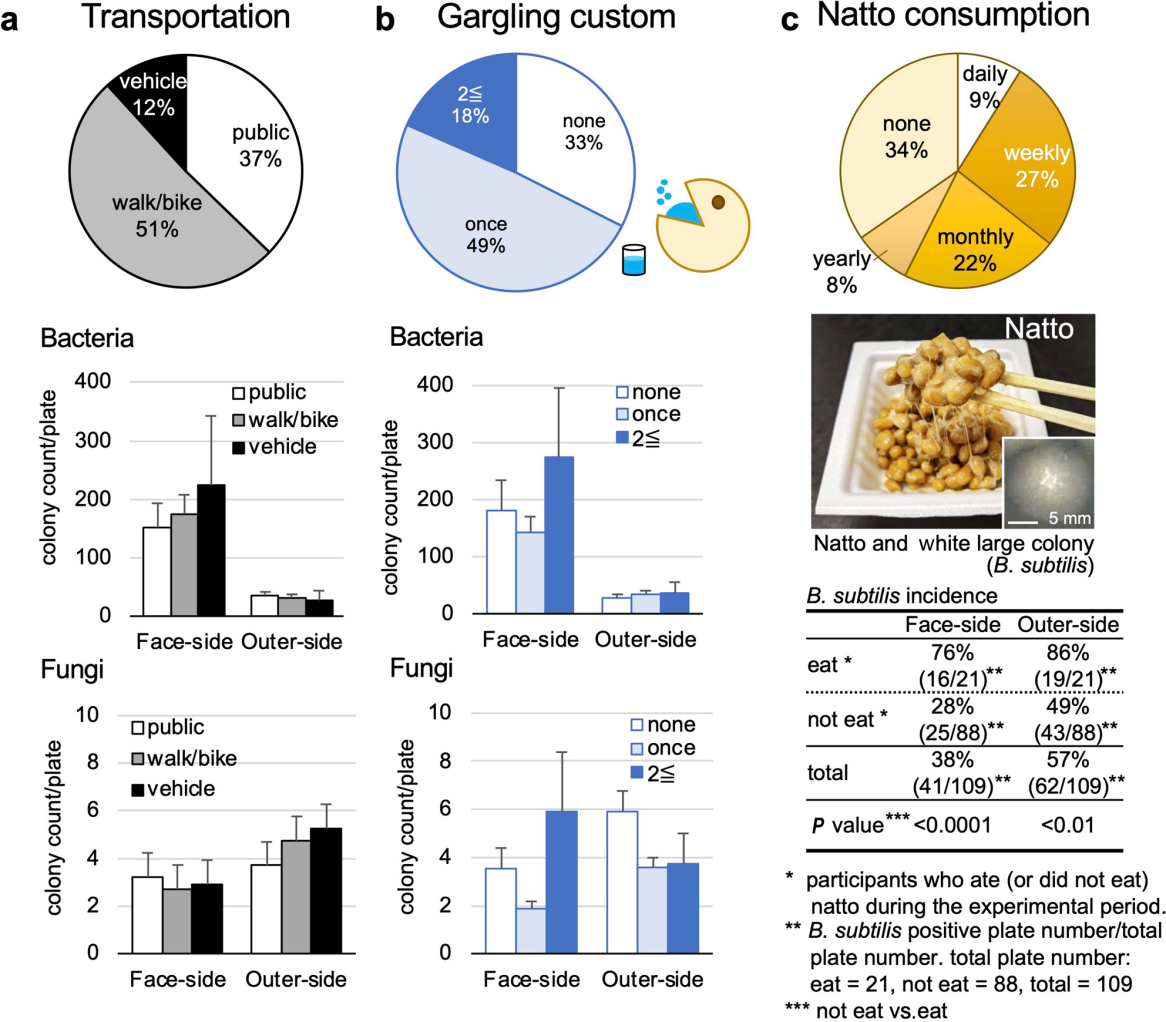
Lifestyles and microbial colonies: transportation, gargling, and natto consumption. (**a**) We categorized three transportation systems to commute: (1) public transportation: trains and/or buses; (2) private vehicles: cars and trucks; and (3) walk/bike: walking, bicycles, and motorbikes. We found no differences in the bacterial and fungal colony counts among the three transportation categories on the face-side or outer-side of masks. (**b**) Microbial colony counts and the gargling habit. The pie chart showed the percentage of participants' gargling frequency; 67% of the participants gargled at least once a day. We found no differences in the bacterial or fungal colony counts among the participants regardless of the gargling frequency. (**c**) Natto consumption and *Bacillus subtilis* colonies. Natto is a traditional Japanese food made from soybeans fermented with *B. subtilis* that forms large white colonies on agar plates. According to the survey, 9% and 27% of the participants have eaten natto daily and weekly, respectively; 19% (21 of 109) of the participants ate natto during the experimental period. The participants who ate natto had a significantly higher percentage of *B. subtilis* colonies than those who did not eat natto.

We determined whether individual lifestyles could affect microbial counts on the masks that originate from the host (i.e., human) or the environment. One of the environmental factors that seemed to affect the levels of microbes on the masks is transportation to commute (Fig. 3a). Here, we classified into three transportation systems: (1) public transportation, including trains and buses; (2) private vehicles such as cars and trucks; and (3) walking, bicycles, and motorbikes. We found no differences in the bacterial or fungal colony counts on both sides of the masks among the three transportation systems.

Next, we evaluated two popular habits in Japan: gargling and natto consumption. Gargling (also known as mouth/throat wash) is a Japanese custom that has been believed to prevent respiratory infections^14^. Of the participants, 67% gargled at least once a day and usually gargled when they returned home. However, there were no differences in the bacterial or fungal colony counts among the participants regardless of gargling (Fig. 3b).

Natto is a traditional Japanese fermented food that is sticky when eaten and clings to the mouth and chopsticks (Fig. 3c). Natto is made by fermenting soybeans with the spore-forming bacterium *Bacillus subtilis*, which can survive dry conditions. As expected, in this study, we observed the large white colonies formed by *B. subtilis*. According to the questionnaire, 9% and 27% of the participants have eaten natto daily and weekly, respectively; 19% of the participants ate natto during the experimental period. The participants who ate natto had a significantly higher incidence of large white *B. subtilis* colonies on both sides of the masks than those who did not.

### Bacterial colony morphologies and identification

**Figure 4:**
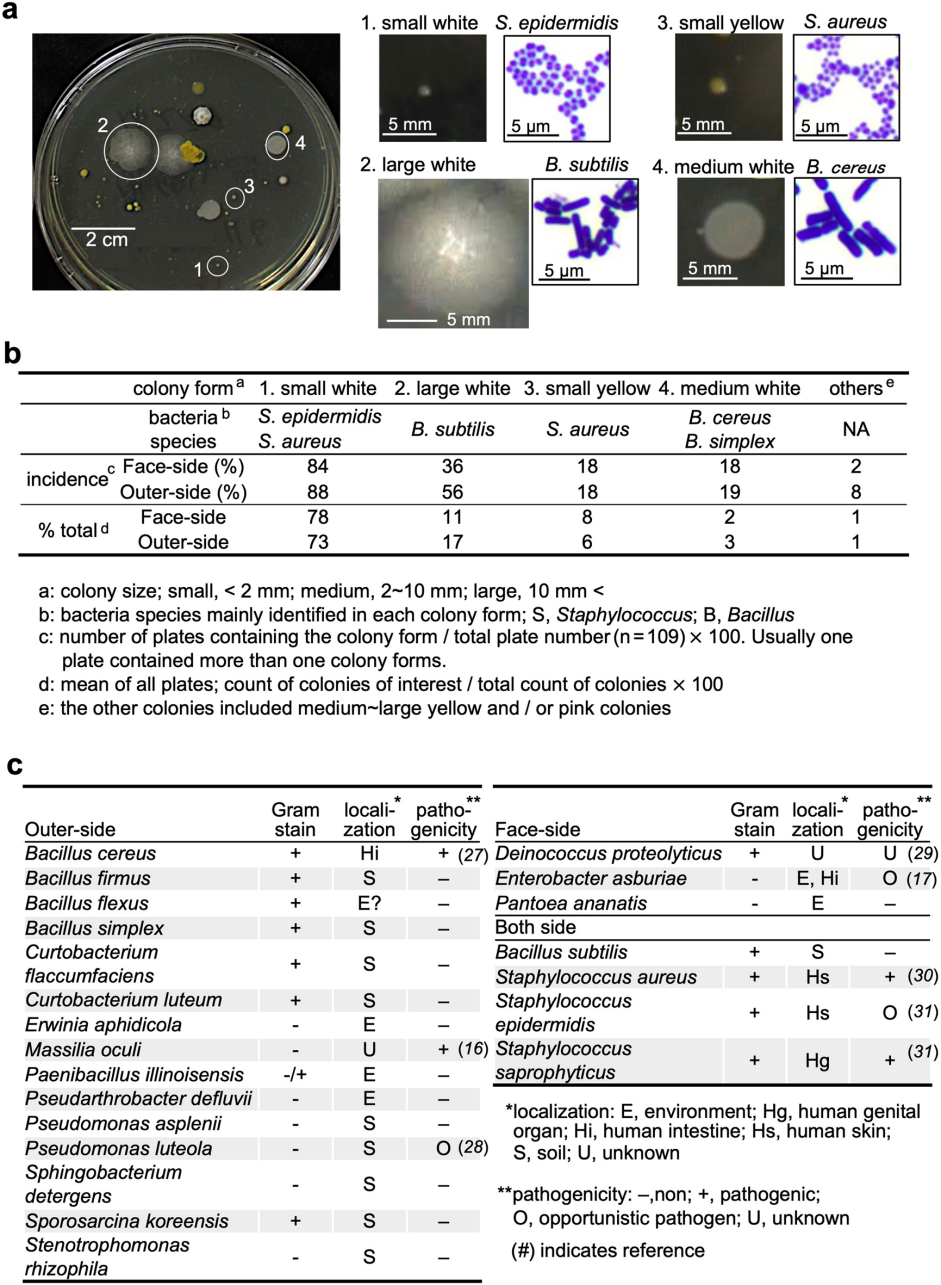
Bacterial colony morphologies and identification. (**a**) We observed a variety of colonies on the agar plates and classified the colonies into four major colony forms, morphologically. Representative bacteria composed of each colony were visualized with their Gram-stain images. (**b**) Major colony forms, identified bacteria, and frequencies (incidence and % total). (**c**) Identified bacteria, their localization, and pathogenicity in humans.

In the bacterial cultures, we observed a variety of colonies on the agar plates (Fig. 4a). We morphologically classified the colonies into four major colony forms and the other forms: (1) small white, (2) large white, (3) small yellow, (4) medium white, and the other forms, including medium to large with yellow or pink, based on the colony size (small < 2 mm, medium 2–10 mm, and large 10 mm <), color, and frequencies (Fig. 4a,b). The frequency of colonies was calculated in two formulas: (I) colony incidence = number of plates containing the colony of interest/total plate number (n = 109) × 100; and (II) % total = counts of colonies of interest/total counts of colonies in each plate × 100 (then, the mean of % total from all plates was calculated). As shown in Fig. 4a, most participants had more than one colony form. The dominance of the four colony forms regarding the colony incidence and mean % total of each colony was overall similar on the face-side and outer-side (Fig. 4b). The small white colonies were most frequently observed, with the incidence and % total exceeding 80% and 70%, respectively.

To further determine the bacteria composing each colony, we conducted Gram staining and 16S ribosomal RNA (rRNA) sequencing. The 16S rRNA sequencing showed that the small white colonies consisted mainly of *Staphylococcus epidermidis,* and/or *S. aureus*; the major bacteria species forming the small yellow colonies was *S. aureus*. The large white colonies were the second most observed ones and consisted of *B. subtilis*, a component of natto (as shown in Fig. 3c). The medium white colonies consisted of *B. cereus* and *B. simplex*; *B. cereus* was identified only on the outer-side of masks. Among the colonies, we also identified other bacterial species by 16S rRNA sequencing (Fig. 4c). Although most identified bacteria were non-pathogenic, there were several potential pathogenic bacteria in humans as follow: *S. aureus* (commensal bacterium, but its overgrowth can cause various diseases); *B. cereus* (intestinal bacterium, causing food poisoning); *Staphylococcus saprophyticus* (urinary tract infection); and *Pseudomonas luteola* (opportunistic pathogen)^15–17^.

### Fungal colonies and identification

**Figure 5:**
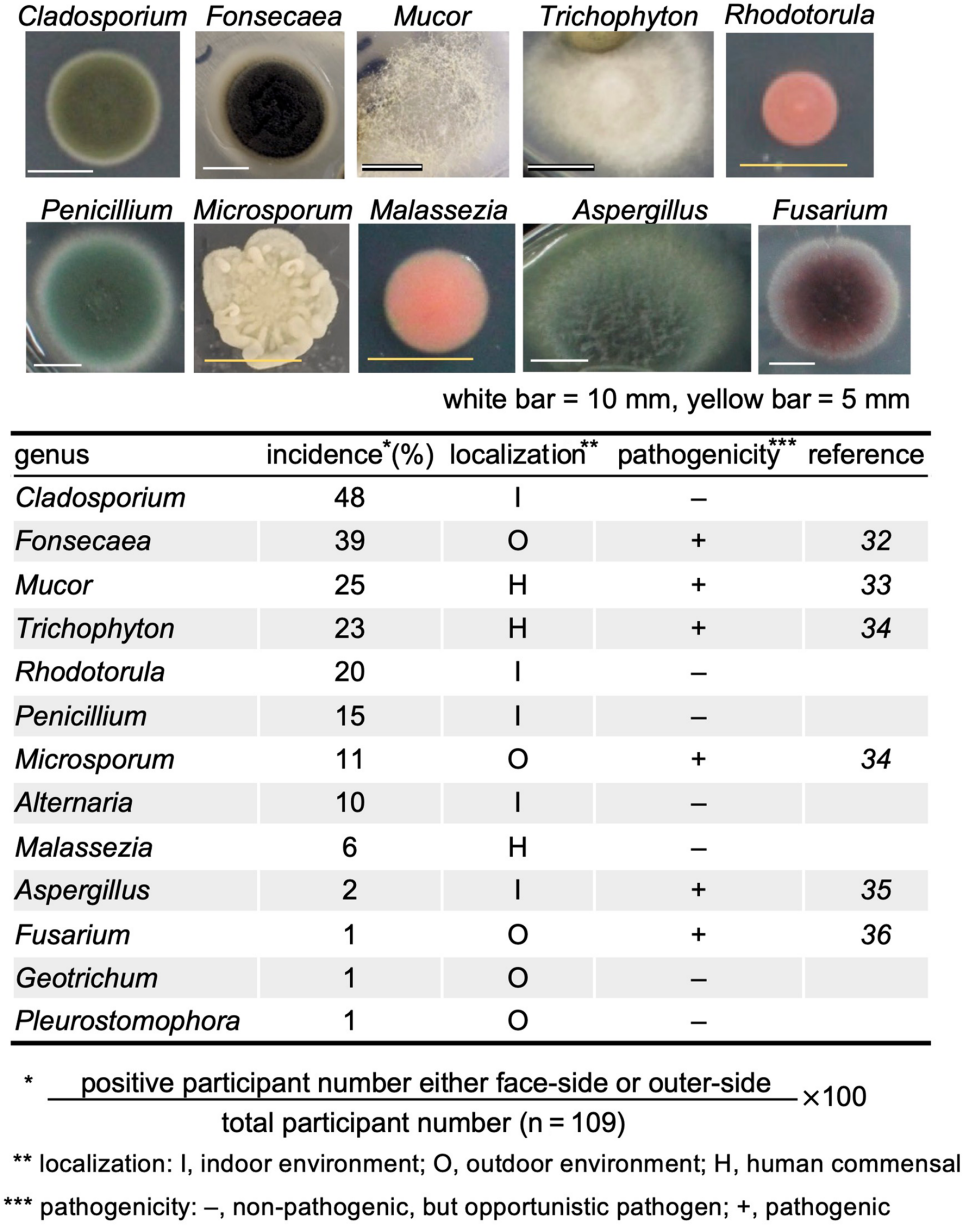
Identification of fungal colonies. We identified fungi by the colony morphology macroscopically as well as the hypha and spore morphology microscopically. Ten representative fungal images were shown. The white and yellow bars are 10 mm and 5 mm, respectively. Identified fungi, the incidence in this study, localization, and pathogenicity were listed.

After quantifying fungal colonies, we further incubated them for another 2 days at 37 °C to induce spore formation. Then, using lactophenol cotton blue staining, we identified fungi on the masks based on the colony morphology macroscopically as well as the hypha and spore morphology microscopically. Although we could not identify some fungi due to lack of spore formation, we identified 13 fungal genera (Fig. 5). Among them, more than 20% of the participants had the four fungal genera, namely *Cladosporium, Fonsecaea, Mucor,* and *Trichophyton*, in common on both sides of the masks. The latter three are potentially pathogenic in humans (Fig. 5).

## Discussion

In this study, we demonstrated the associations between several factors and microbial contaminations of face masks commonly used worldwide during the COVID-19 pandemic. Although some of our findings were what we had anticipated, there were several unpredicted findings, which need to be addressed as essential hygiene issues. In Table 1, we summarized the major findings and showed the results with statistical differences in bold (*P* < 0.05). The colony counts of face masks were higher in bacteria than in fungi; the bacterial and fungal colony counts were higher on the face-side and outer-side, respectively. The longer duration of mask usage correlated with increases in the fungal colony counts but not the bacterial colony counts. We also found that non-woven masks had fewer fungi than other mask types on the outer-side. Although the bacterial colony counts were comparable in all mask types, those on the face-side were lower in females than in males.

**Table 1:** Factors associated with microbial colony counts on face masks.

	Bacteria	Fungi
Colony count/plate	1–1600	1–22
Face-side/outer-side	**High on the face-side**	**High on the outer-side**
Duration of usage	No effect	**High in 2 days ~ **
Mask type	No effect	Low in non-woven outer-side
Gender	**Low in female (face-side)**	**High *Cladosporium* in female**

Boldface indicates a significant difference (*P* < 0.05).

We further conducted a receiver operating characteristic (ROC) analysis to see the associations among the data obtained in this study shown in Table 2, where the area under the curve (AUC) indicated positive and negative associations (Figs. 2e, S1). The genus *Cladosporium*, the most frequently detected fungus in this study, was more frequently detected in females (58% females and 29% males). *B. subtilis* was more frequently detected on the masks used by the participants who ate natto at least once a month. In contrast, the transportation systems were not associated with bacteria or fungi colony counts. These results were consistent with our findings in Fig. 3, where neither public transportation usage nor gargling altered the bacterial or fungal colony counts. On the other hand, eating natto strongly increased the *B. subtilis* colony counts on the masks. Although *B. subtilis* multiplies rapidly and forms colonies large enough to outcompete other bacterial colonies, the presence of *B. subtilis* did not affect the counts of *S. epidermidis*, the most frequently detected bacterium in this study. The counts of white medium colonies seemed to be negatively affected by the presence of *B. subtilis* (AUC = 0.65). This is consistent with the previous report^18^ that *B. subtilis* inhibited the growth of *B. simplex*, which was a major component of a medium-sized white colony in the current study.

**Table 2:** Receiver operating characteristic (ROC) analysis.

Factor	Variable	AUC	Association
Mask type, non-woven	Outer-side fungal count	**0·77**	Negative*
Gender, female	Face-side bacterial count	**0·71**	Negative†
Usage $\geqq$ 2 days	Outer-side fungal count	**0·65**	Positive*
Gender, female	*Cladosporium* positive	**0·65**	Positive
*B. subtilis*, inside	White medium colony	**0·65**	Negative
Natto$\geqq$ once/month	*Bacillus subtilis*	**0·61**	Positive
Public transportation	Bacterial or fungal count	0·50	No
*B. subtilis*, inside	*Staphylococcus epidermidis*	0·42	No

Boldface shows AUC higher than 0.6

AUC: 0.5–0.6, unsatisfactory; 0.6–0.7, satisfactory; 0.7–0.8, good; 0.8–0.9, very good; 0.9–1, excellent.

*, ^†^Associations were consistent with statistical differences shown in *, Figs. 2; †, S1.

Most fungi isolated in this study were opportunistic pathogens rather than pathogenic (Fig. 5), although immunocompromised hosts should be advised to wear non-woven masks on a daily basis. We detected *B. cereus*, a foodborne pathogen, on the outer-side of masks in 5% of the participants (Fig. 4c), suggesting that *B. cereus* might adhere to the face masks through hands from feces. Intensive handwashing is recommended, since handwashing is effective in reducing the incidence of diarrhea^19^.

Although we anticipated that the counts of bacterial colonies could increase due to the duration of mask usage, this was not the case. The moisture requirement of bacteria may explain this^20,21^. While we wear a face mask, the humidity under the mask space becomes approximately 80%, in which bacteria can survive and grow^22,23^. In contrast, when a used mask is not worn for a long time, particularly at night, it dries out overnight and bacteria on the mask are likely to die due to the dry conditions. On the other hand, since fungi and their spores are resistant to drying, they can survive under the condition where masks dry out. This explains why fungi tended to accumulate and increase with longer mask usage. When we compared the microbial colony counts between the mask types, there were no substantial differences in the microbial colony counts between non-woven and other mask types. These findings suggest that the higher fungal colony counts on the outer-side of masks would be due to the duration of mask usage, but not the mask types. Regarding washable/reusable masks (“other types” of masks in the current study), the proper cleaning method for cotton face masks has been recommended to reduce the microbial load on the masks^12^. However, in the current experiments, we did not find significant differences in bacterial or fungal colony numbers on the masks based on washing (Fig. S2). This could be explained by lack of information about the proper cleaning method for most mask users (i.e., boiling at 100 °C, washing at 60 °C, or ironing with a steam iron) to disinfect the masks.

There were a few studies reporting microbial isolation on masks; a Belgian group investigated bacterial colony numbers on face masks in experimental settings, where 13 volunteers wore cotton and surgical masks for 4 h^12^. The authors harvested bacteria by vortexing the masks (without separation into the face-side and outer-side layers) with PBS and cultured the bacteria on the brain heart infusion (BHI) and lysogeny broth (LB) agar plates. They found that the bacterial colony number was higher in the cotton masks than in the surgical masks and that the major bacterial genera from the surgical masks were *Staphylococcus* and *Streptococcus*. Our study also detected *Staphylococcus*, but not *Streptococcus* that cannot grow on the BHI plate.

The bacterial colony counts on the face masks were higher in males than in females among the daily users (Fig. S1). We suspected that the difference could be associated with a more intensive facial skincare by females than by males. Thus, we performed a principal component analysis (PCA), using the survey data based on a daily facial skincare routine (three categories: 1. face wash method, 2. lotion/sunscreen usage, and 3. foundation usage) as well as the bacterial and fungal colony counts of masks worn for 4 h (Fig. S3a). The proportion of variance of principal component (PC) 1 was 44%; PC1 values reflected more intensive facial skincare. Here, the bacterial colony numbers and three skincare categories contributed negatively and positively to PC1 values, respectively. This suggested that more intensive facial skincare may decrease bacteria on the face masks. Among the three skincare categories in the survey, we tested whether the foundation usage could affect the number of bacterial colonies. We recruited volunteers and asked them to wear the mask for 4 h with foundation applied to only the left half of their faces. We found no differences in the bacterial colony numbers between the left and right halves of the face masks (Fig. S3b). Furthermore, neither lotion/sunscreen usage nor the face wash method statistically decreased the bacterial colony numbers by itself (data not shown). Although we did not examine other factors that may contribute to the gender difference in the bacterial colony counts, the potential factors include the higher facial temperature in males^24^ and the gender difference in sweat and sebum^25^.

There were several limitations in this study. First, the survey of face masks in this study was not comprehensive, and the sample size was small. Although the face masks were classified into three major types, they can be further subdivided according to the thickness, fabric coating, and other factors that may affect microbial growth. In experimental settings, the bacterial colony number and composition differed between surgical and cotton face masks after 4-h of wearing^12^. Second, in all the experiments, since the face masks were put on and taken off with bare hands, there was a possibility that microbes on the hands could be transferred to the face masks. Here, we intentionally instructed the participants not to wear gloves during the experimental period, since our objectives were to examine bacteria and fungi on the face masks under our normal lifestyles. Microbial colonies detected from new non-woven masks handled with bare hands were negligible (average 6.5 bacterial and no fungal colonies, data not shown). Lastly, there is an argument that the face masks need to be thoroughly washed with detergent broth for better isolation of microbes on masks^26^. In this study, however, we decided to collect microbes on the face masks by simply pressing them onto agar plates. Although this method may leave substantial microbes on the mask materials, we believe that easily detachable microbes are more relevant to respiratory infections.

In this study, we focused on a newly emerged-hygiene issue in the current lifestyles of wearing face masks during the COVID-19 pandemic. These results will provide new insights into face mask usage to prevent potential pathogenic infections.

## Methods

### Mask layer imaging

A non-woven mask was composed of three layers, each of which was cut with scissors and separated manually. A gauze mask was composed of multiple layers, one of which was separated manually. We directly placed a polyurethane mask (without sample preparation) or each layer of the non-woven and gauze masks on the microscope stage of the CX33 Microscope (Olympus, Tokyo, Japan) and imaged using 10 × objective lens with the CCD Camera DP22 (Olympus).

### Study design

This study was conducted between September and October 2020. The participants were 109 medical students, 63 males (aged 22.4 ± 0.4) and 46 females (aged 21.2 ± 0.3, no significant difference between genders) at Kindai University Faculty of Medicine, Osaka, Japan. All experimental protocols were approved by the Institutional Biosafety Committee of Kindai University and performed by the institutional guidelines. Informed consent was obtained from all participants. The survey for the participants was as follows: age, gender, type of mask, duration of mask usage, transportation, gargling habit, and natto consuming habit. We confirmed that no participants were treated with antimicrobial drugs during the experimental periods.

### Sample collection, microbial culture, and colony count

To isolate and culture the microbes adhered to face masks, the face-side and outer-side of the face masks were pressed onto agar plates (8.6 cm in diameter, 58 cm^2^ in area), separately, which were covered with the lids immediately to avoid contamination. The culture conditions were as follows: for the bacterial cultures, BHI agar plates (Eiken chemical Co., LTD, Tochigi, Japan) or Soybean-casein digest broth with lecithin and polysorbate 80 (SCDLP) agar plates (Eiken chemical Co., LTD,) were used and incubated at 37 °C under the aerobic condition for 18 h. We found similar colony numbers and morphology between the BHI and SCDLP agar plates. This is consistent with the previous findings reported by Delanghe et al., where the bacterial colony numbers from surgical mask samples were comparable between the BHI and LB agar plates^12^. Thus, in all subsequent experiments, we decided to use BHI agar plates, which are widely used as a general-purpose growth medium. In the longer incubation (> 2 days), the fast-growing bacterium *B. subtilis* outgrew the other bacteria, resulting in the difficulty of detecting slow-growing bacteria. For the fungal cultures, Sabouraud dextrose agar plates (Nissui pharmaceutical Co., LTD, Tokyo, Japan) were used and incubated at 25 °C under aerobic condition for 5 days. Following the primary incubation, we evaluated the colony morphology and conducted colony counting. Although we tested the presence of microbes on the middle layer (filter layer), we detected only small numbers of the bacterial and fungal colonies (mean ± SEM: bacterial colonies, 6.3 ± 4.9; and fungal colonies, 1.0 ± 0.5). Thus, we decided to focus on the microbial colonies on the face-side and outer-side of the masks in this study.

### Identification of microbial colonies

Bacteria: we collected 94 colonies from the cultured plates, isolated DNA, and conducted 16S ribosomal RNA (rRNA) sequencing by the MiSeq (Illumina, San Diego, CA) at the Center for Oral Microbiota Analysis (Takamatsu, Japan). We also prepared bacterial smears on glass slides for Gram-staining (Fujifilm Wako, Osaka, Japan) and took the microscopic images using the CX33 Microscope with the CCD Camera DP22.

Fungi: we selected representative agar plates containing different types of fungal colonies from all cultured plates. We further incubated the cultured plates at 37 °C for 2 days to induce the spore formation, stained the fungi with lactophenol cotton blue (Muto pure chemical Co., LTD, Tokyo, Japan), and identified them based on their colony morphology and microscopically^37^.

### Data analyses

We conducted PCA using the software RStudio (version 1.4.1106) and Exploratory (Exploratory, Inc., CA). For statistical analyses, we conducted the paired *t*-test, Student’s *t*-test, and χ^2^ test. To determine the correlations between the data obtained in this study, we conducted an ROC analysis to evaluate the association between the factors and outcomes by calculating the AUC. The AUC close to 1 indicates a strong association, and less than 0.5 indicates no association.

## Supplementary Information

Supplementary Information.

## Data Availability

The datasets generated and/or analyzed during the current study are available from the corresponding author on reasonable request.
