# Supplementary material for: Bacterial and fungal isolation from face masks under the COVID-19 pandemic
Source: Sci Rep. 2022 Jul 18;12:11361. doi: 10.1038/s41598-022-15409-x (PMC9293923; doi:10.1038/s41598-022-15409-x)
Supplement: Supplementary file 1 — Supplementary Information. [file 41598_2022_15409_MOESM1_ESM.pdf]

Supplementary figures 1 – 3

Title: Bacterial and fungal isolation from face masks under COVID-19 pandemic

Authors; Ah-Mee Park*, Sundar Khadka, Fumitaka Sato, Seiichi Omura, Mitsugu Fujita, Kazuki Hashiwaki, Ikuo Tsunoda.

Affiliation; department of Microbiology, Kindai University Faculty of Medicine, Osakasayama, Osaka, Japan

*Corresponding author; ampk@med.kindai.ac.jp

Supplementary figure 1

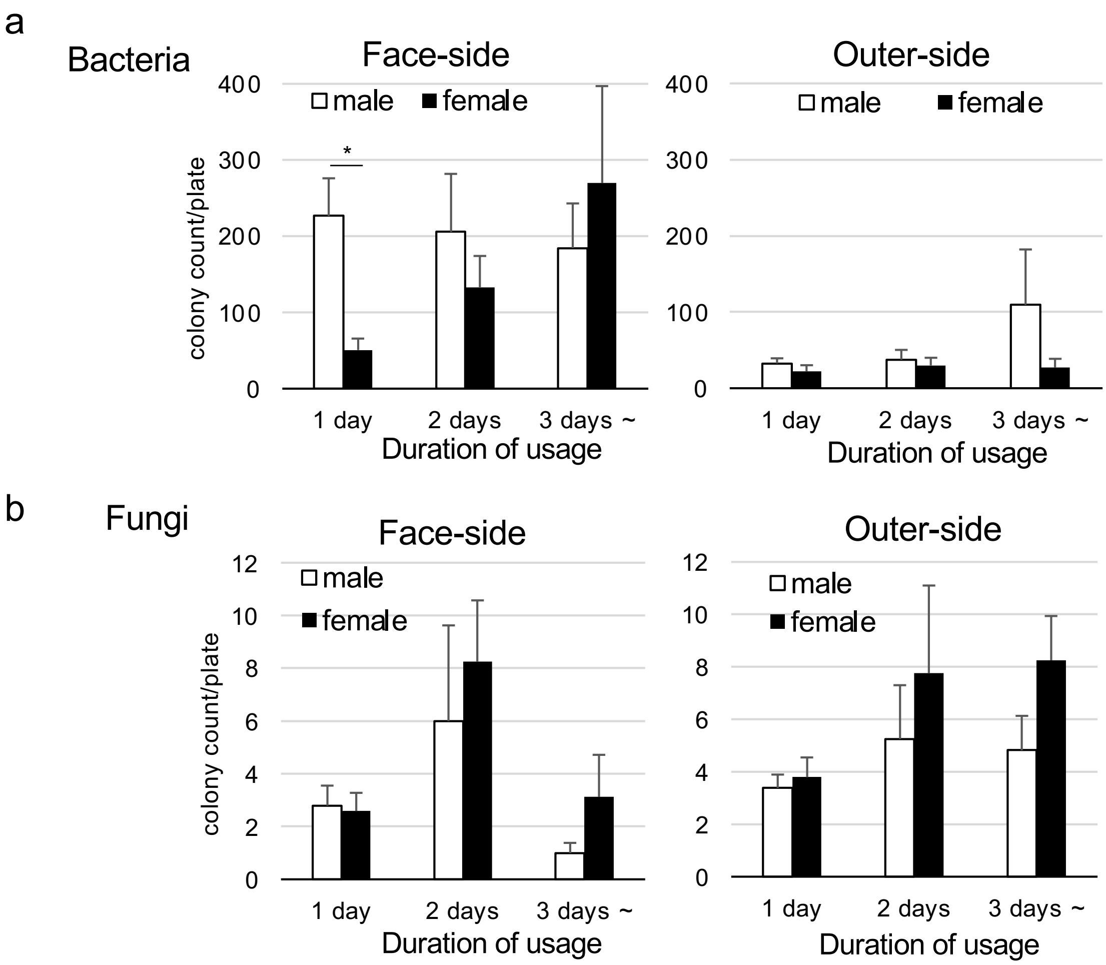

Supplementary figure 1. Bacterial **(a)** and fungal **(b)** counts were compared based on the duration of usage and gender, mean + SEM. **P*<0.05. Student's *t*-test was used for statistical analyses.

Supplementary figure 2

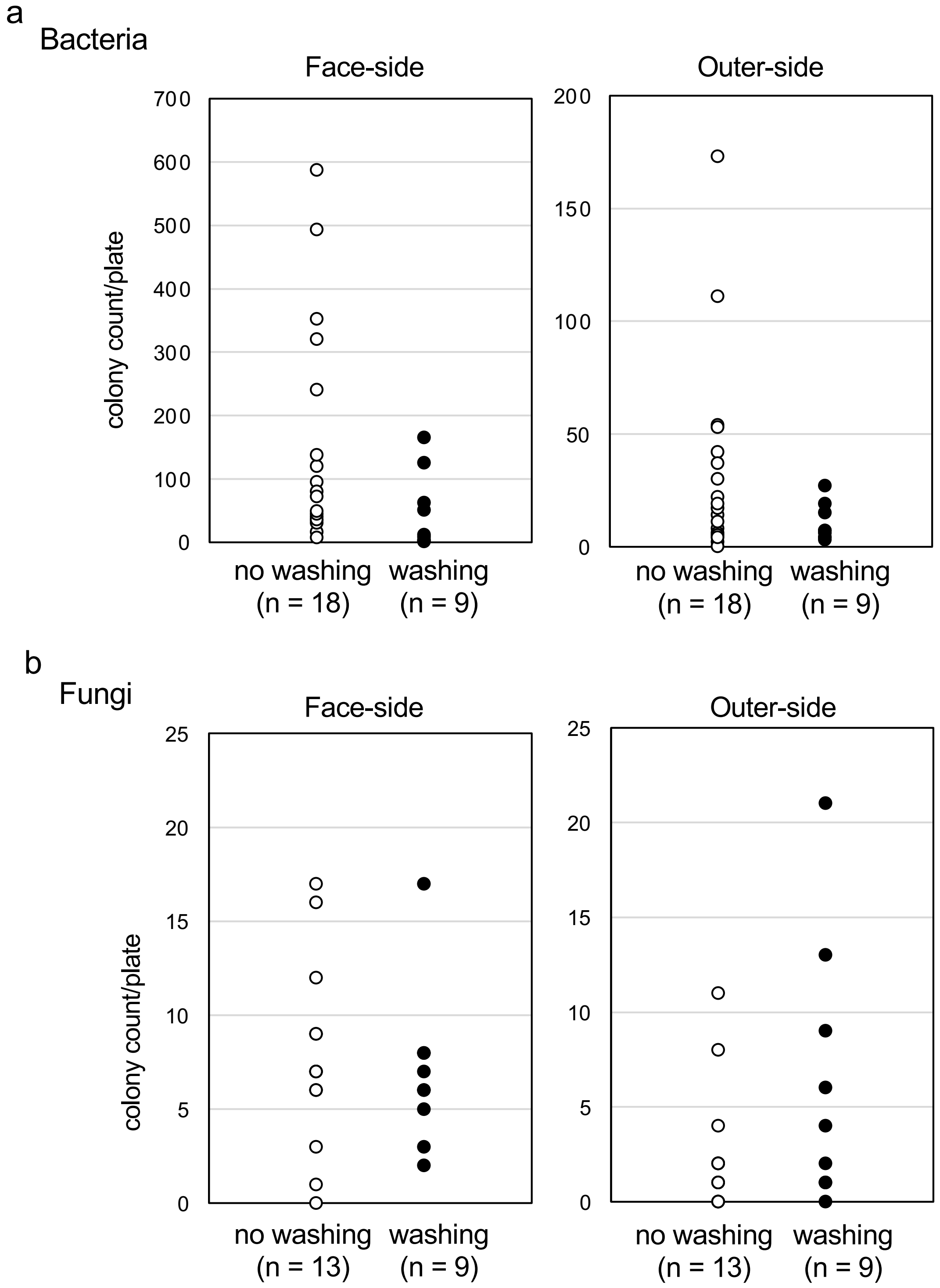

Supplementary figure 2. Bacterial (**a**) and fungal (**b**) colony counts were compared in reusable mask users based on washing. There was no significant difference between two groups.

Supplementary figure 3

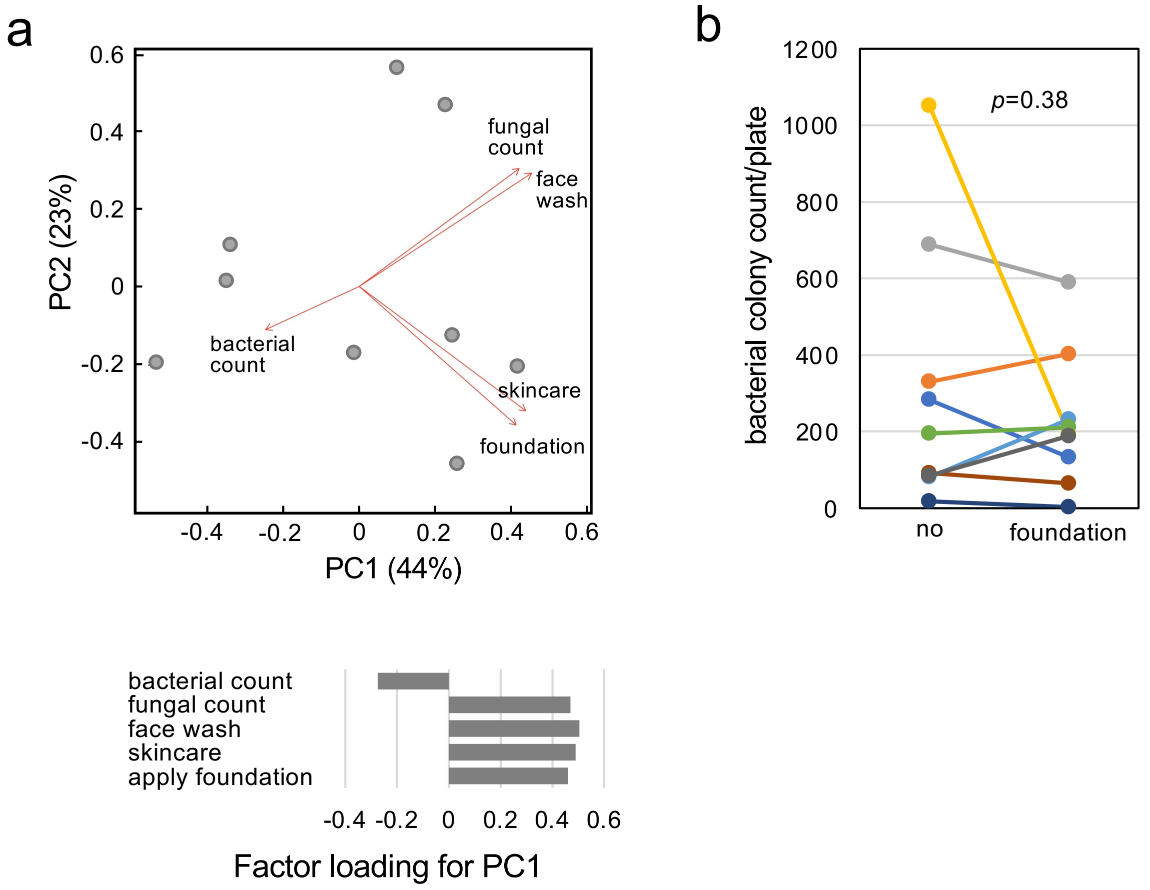

Supplementary figure 3. Skincare and mask microbes. **a.** Nine participants wore non-woven masks for 4 hours and the face-side bacteria and the outer-side fungi were cultured, then each colony count was counted. Principal component analysis (PCA) was performed by using the count of each microbe colony and skincare-related survey results. Survey scorings are as follow:

face wash: 1 = no, 2 = without detergent, 3 = with detergent

skincare: 1 = none, 2 = skin and milk lotion, 3 = skin and milk lotion + sunscreen

foundation: 1 = none, 2 = use

b. Nine participants (three males and six females) applied foundation to the left half of their face and wore a non-woven mask for 4 hours. All volunteers used Mineral Moist Cream®, whose major components include water, butylene glycol, and octyl 4-methoxycinnamate without preservative paraben (Azuma Shoji Co., Ltd., Osaka, Japan). Bacteria adhering to the face-side of masks were cultured for 18 hours on agar plates. Bacteria derived from the left half (no) and right-half (foundation) were cultured separately and the colony numbers were counted.
